# Gambling-Specific Cognitions Are Not Associated With Either Abstract or Probabilistic Reasoning: A Dual Frequentist-Bayesian Analysis of Individuals With and Without Gambling Disorder

**DOI:** 10.3389/fpsyg.2020.611784

**Published:** 2021-01-26

**Authors:** Ismael Muela, Juan F. Navas, José C. Perales

**Affiliations:** ^1^Department of Experimental Psychology, Mind, Brain, and Behavior Research Center (CIMCYC), Universidad de Granada, Granada, Spain; ^2^Department of Clinical Psychology, Complutense University of Madrid, Madrid, Spain

**Keywords:** gambling-related cognitions, abstract reasoning, probabilistic reasoning, intelligence, motivated reasoning, gambling disorder

## Abstract

**Background:**

Distorted gambling-related cognitions are tightly related to gambling problems, and are one of the main targets of treatment for disordered gambling, but their etiology remains uncertain. Although folk wisdom and some theoretical approaches have linked them to lower domain-general reasoning abilities, evidence regarding that relationship remains unconvincing.

**Method:**

In the present cross-sectional study, the relationship between probabilistic/abstract reasoning, as measured by the Berlin Numeracy Test (BNT), and the Matrices Test, respectively, and the five dimensions of the Gambling-Related Cognitions Scale (GRCS), was tested in a sample of 77 patients with gambling disorder and 58 individuals without gambling problems.

**Results and interpretation:**

Neither BNT nor matrices scores were significantly related to gambling-related cognitions, according to frequentist (MANCOVA/ANCOVA) analyses, performed both considering and disregarding group (patients, non-patients) in the models. Correlation Bayesian analyses (bidirectional BF_10_) largely supported the null hypothesis, i.e., the absence of relationships between the measures of interest. This pattern or results reinforces the idea that distorted cognitions do not originate in a general lack of understanding of probability or low fluid intelligence, but probably result from motivated reasoning.

## Introduction

Gambling is a leisure activity, practised non-problematically by a large share of the population, but that can generate substantial harm to the community (Shannon et al., 2017). The severity of potentially problematic gambling lies on a continuum in which gambling disorder is placed at its highest end (Shaffer and Martin, 2011; Rai et al., 2014). However, from a public health perspective, gambling-related harms go beyond the individual, and are not exclusively driven by the severity of disordered gambling (Wardle et al., 2019).

Understanding the factors that foster gambling involvement is thus important at the individual, social, and policy levels, regardless of clinical status. And, among these factors, distorted gambling-related cognitions play a central role (Fortune and Goodie, 2012; Lindberg et al., 2014a; Goodie et al., 2019; Brooks et al., 2020). These cognitions are frequently targeted by commercial advertising (Lopez-Gonzalez et al., 2018), and are among the main therapeutic targets in cognitive-behavioral therapy of gambling disorder (Rash and Petry, 2014; Choi et al., 2017; Menchon et al., 2018). Indeed, they are present to some degree in virtually all gamblers, play a key role in maintaining gambling behavior [see (Goodie and Fortune, 2013), for a review], and their strength varies as a function of severity (Emond and Marmurek, 2010; Del Prete et al., 2017; Jara-Rizzo et al., 2019) and is modulated by the effectiveness of therapy (Breen et al., 2001; Doiron and Nicki, 2007; Toneatto and Gunaratne, 2009; Donati et al., 2018).

The most comprehensive and widely used model of gambling-related cognitions [the Gambling-Related Cognitions Scale, GRCS (Raylu and Oei, 2004)], encompasses five different domains, namely, *inability to stop, expectancies, predictive control, illusion of control*, and *interpretative bias*. The first two are common dysfunctional (but not necessarily “erroneous”) beliefs present in a range of potentially addictive behavior patterns. Specifically, inability to stop refers to a lack of self-efficacy in controlling gambling behavior and overcoming urges, and expectancies allude to expected outcomes than can work as motives to gamble, such as winnings or curbing negative affect. The other three can be strictly considered cognitive biases at making causal inferences. Illusion of control and predictive control are beliefs about the possibility to control and predict gambling outcomes, respectively. Interpretative bias is the tendency to attribute positive and negative gambling outcomes to internal and external causes, respectively, that is, to reformulate wins as due to skills, and losses as due to bad luck (Oei and Burrow, 2000; Oei and Raylu, 2004).

There are at least two mechanisms by means of which better domain-general reasoning abilities could protect individuals from distorted gambling cognitions, and thus, indirectly, from developing gambling problems. The first one is more specific: given the evident overlap between poor understanding of probability and randomness, and causal biases (Gilovich et al., 1985; Ladouceur et al., 1996; Clark, 2017), it seems reasonable to assume that people with lower scores in probabilistic reasoning will transfer that disadvantage to gambling activities, where, as mentioned earlier, causal misattribution plays a key role. Or the other way round, good domain-general probabilistic reasoning could potentially prevent the development of at least some types of distorted gambling-related cognitions.

The second mechanism is more general, and regards the potential role of general fluid intelligence and abstract reasoning. These two largely overlapping constructs refer to the capacity to think logically, solve novel problems and operate abstract symbols with minimal dependence on previously acquired knowledge (Carpenter et al., 1990; Santarnecchi et al., 2017; Gómez-Veiga et al., 2018). Gambling devices and the rules under which they operate can be mathematically complex and opaque, so, in principle, fluid intelligence could contribute to a better understanding of how gambling devices work, and thus to override cognitive biases (Evans and Over, 2010). Complementarily, fluid intelligence could foster a more reflective reasoning style (Barrouillet, 2011), and thus preclude the tendency to rely on the device-triggered intuitions and heuristics from which gambling-related cognitions seem to originate.

Nonetheless, the possibility that gambling-related cognitions (and specifically gambling-related biases) could be disconnected from general reasoning abilities has also been theoretically articulated. In some previous work, it has been shown that dysfunctional gambling-related cognitions, and especially gambling-related causal biases and misattributions, as measured by the GRCS, are more prevalent in individuals playing skill-based games, who, in turn, tend to be younger and better educated, relative to individuals who mostly practice pure chance games (Griffiths et al., 2009; Myrseth et al., 2010; Wood and Williams, 2011). In the context of the Gambling Space Model [GSM, (Jara-Rizzo et al., 2019; Navas et al., 2019; Ruiz de Lara et al., 2019)], more dysfunctional cognitions and stronger gambling-related biases are not hypothesized to originate in weaker domain-general reasoning processes, but in domain-specific motivated reasoning. This kind of reasoning (Kunda, 1990) is driven by ego-protection, that is, it is used by the individual to disguise the real (and potentially ego-damaging) reasons that drive gambling, to make gambling more acceptable, and to reappraise aversive gambling outcomes. In other words, the underpinnings of gambling cognitions would not be mainly intellectual, but affective (Navas et al., 2016, 2017b).

### A Brief Review of the Literature on the Link Between Domain-General Reasoning and Gambling Cognitions

Studies on domain-general reasoning skills in gamblers fall into three broad categories. In the first one, intelligence or domain-general reasoning is recorded only for control purposes, in case-control designs with problematic vs. non-problematic gambling (so that domain-general reasoning measures were not the main variables of interest). This category is heterogeneous and the studies in it do not systematically report associations between domain-general reasoning and gambling cognitions. With regard to the association between domain general reasoning and gambling disorder symptoms or diagnosis, results are mixed: in some studies, the group with disordered or problematic gambling obtained lower scores than controls in domain-general reasoning constructs (Martínez-Pina et al., 1991; Toplak et al., 2007; Forbush et al., 2008), whereas, in others, the groups did not show significant differences (Brevers et al., 2012). It is important to take into account, however, that in part of these studies, domain-general reasoning scores were intentionally matched across groups (groups were sampled *a priori* to show no differences in general reasoning ability), so the absence of differences in reasoning abilities between groups is not always informative. For that reason, studies in which matching in general reasoning measures was forced are not included in this review.

A second category of studies has intentionally investigated the putative associations between gambling severity (or presence of gambling disorder/problem gambling) and domain-general reasoning (Templer et al., 1993; Fernández-Montalvo et al., 1999; Delfabbro et al., 2006; Lambos and Delfabbro, 2007; Kaare et al., 2009; Hodgins et al., 2012; Rai et al., 2014; Primi et al., 2017) in broad community or convenience samples, using regression or correlation techniques. These show that individuals with low domain-general reasoning abilities show more severe gambling problems or are in a higher risk of presenting disordered or problematic gambling, with few exceptions [(Fernández-Montalvo et al., 1999); in Primi et al. (2017), gambling problems’ severity was found to correlate positively with fluid intelligence, but negatively with probabilistic reasoning]. Again, however, gambling-specific cognitions were not central variables of interest. With the exception of Lambos and Delfabbro (2007), the moderating role of gambling-related cognitions in the association between general reasoning and gambling problems was not assessed either.

Studies of these two categories, primarily or supplementarily estimating the association between domain-general reasoning abilities and presence or severity of gambling problems, are summarized in Table 1.

**TABLE 1:** Characteristics and summary of results of the revised studies on the relationship between domain-general reasoning abilities and gambling symptoms’ severity.

Study	*N*	Participants	Severity index	Domain-general reasoning task	Main findings regarding severity/diagnosis of gambling disorder and domain-general reasoning
Brevers et al. (2012)*	100	27 PG 38 PrG 35 HC	SOGS	WAIS Vocabulary and WAIS Block Design	PGs, PrGs and controls were similar in estimated IQ. Groups were not intendedly matched in IQ *a priori*
Delfabbro et al. (2006)^†^	926	Approximately, 5% of the sample were PrG. The rest were non-PrG	DSM-IV-J criteria for PG in children and VGS	Five questions about understanding of odds and probabilistic concepts	Little evidence that PrGs had a poorer understanding of the objective odds of gambling activities. PrGs were more accurate than non-PrG on one question concerning binary odds
Fernández-Montalvo et al. (1999)^†^	69	69 PG	SOGS	Raven’s Standard Progressive Matrices	Non-significant negative correlation between fluid intelligence and SOGS
Forbush et al. (2008)*	59	25 PG 34 HC	SOGS	WAIS Letter-Number Sequencing and WAIS Picture Completion	PGs performed significantly worse than controls on the two WAIS subtests
Hodgins et al. (2012)^†^	136	60 PrG 76 non-PrG	CPGI (frequency). PGSI and CIDI (severity)	WASI Vocabulary and WASI Matrix reasoning	PrGs performed significantly worse than non-PrGs on intelligence subtests
Kaare et al. (2009)^†^	75	33 PG 42 NG	SOGS (compared with DSM-IV criteria for PG)	Raven’s Standard Progressive Matrices	PGs had significantly lower total scores than controls in fluid intelligence. Low cognitive ability was among the main predictors of pathological gambling, they but remained non-correlated with gambling-related irrational beliefs
Lambos and Delfabbro (2007)^†^	135	44 PG 46 RG 45 IG	SOGS	Numerical Reasoning Ability and five questions about understanding of odds	There was no significant difference between the groups for their knowledge of gambling odds. PGs and RGs had significantly lower total scores than IGs for numerical reasoning ability
Martínez-Pina et al. (1991)*	172	57 PG 115 HC	SOGS	WAIS	Intelligence was lower in PGs than in controls
Primi et al. (2017)^†^	822	822 students	SOGS	Advanced Progressive Matrices and PRS	Significantly positive correlation between SOGS and fluid intelligence, and significantly negative correlation between SOGS and probabilistic reasoning
Rai et al. (2014)^†^	7461	36 PrG 4557 non-PrG 2234 NG	DSM-IV diagnostic criteria for PG	NART Verbal IQ	PrGs had a significantly lower estimated verbal IQ than non-PrGs and non-gamblers. The odds of PrG nearly doubled with each 1 SD drop in IQ
Templer et al. (1993)^†^	136	136 men convicted	SOGS	Raven’s Standard Progressive Matrices	Higher gambling scores were associated with more unfavorable scores on fluid intelligence
Toplak et al. (2007)*	107	24 PG 26 risk non-PG 57 non-PrG	SOGS and DSM-IV diagnostic criteria	WAIS-R Vocabulary and WAIS-R Block Design	PGs and subclinical gamblers tended to have significantly lower WAIS-R scores than non-PrGs

PG,Individuals with pathological gambling; PrG,Individuals with problem gambling; IG,Infrequent gamblers; RG,Regular Gamblers; HC,Healthy controls; NG,Non-gambling individuals; SOGS,South Oaks Gambling Screen; CPGI,Canadian Problem Gambling Index; VGS,Victorian Gambling Screen; PGSI,Problem Gambling Severity Index; WAIS,Wechsler Adult Intelligence Scale; WASI,Wechsler Abbreviated Scale of Intelligence; PRS,Probabilistic Reasoning Scale; NART,National Adult Reading Test. *Studies in which domain-general reasoning was recorded only for control purposes, in between-participants designs with problematic vs. non-problematic gambling. ^†^Studies primarily investigating the associations between gambling severity (or presence of gambling disorder/problem gambling) and domain-general reasoning (see section “A Brief Review of the Literature on the Link Between Domain-General Reasoning and Gambling Cognitions”). None of the studies from our research team is included in this table, due to partial sample overlapping with the present one.

A third category of studies, more directly relevant to the aims of the present study, has directly investigated whether gambling-related cognitions are underpinned in some way by domain-general reasoning processes. Most of the studies in this category are also observational or correlational, but they do straightforwardly focus on the relationship between domain-general and gambling-related reasoning. For instance, using a card-guessing task, Xue et al. (2012a) found that students with higher cognitive abilities (intelligence and executive function) were more prone to show the gambler’s fallacy. i.e., the erroneous belief that streaks of bad luck are bound to end in a win. In a similar vein, Perales et al. (2017) found gamblers with stronger biases to perform better than gamblers with weaker biases on non-gambling related causal learning tasks [for a different, although compatible, result, see Orgaz et al. (2013)]. The abovementioned study by Lambos and Delfabbro (2007), beyond the association between gambling problems and general understanding of odds, also found such a measure of odds understanding to be unpredictive of gambling-related irrational beliefs. However, in a recent study by Delfabbro et al. (2020), participants who reported greater illusory control in non-gambling-related everyday tasks (in a self-report questionnaire) scored higher on standardized measures of gambling-specific illusory control.

To our knowledge, only one study in this last category has directly intervened on general-domain reasoning abilities in an attempt to reduce gambling-related biases. Donati et al. (2018) showed that a preventive intervention to modify erroneous cognitions by shaping probabilistic and superstitious thinking in adolescents, reduced their erroneous gambling-related cognitions, suggesting that gambling-related cognitions could related to domain-general reasoning.

### Present Study

The present study is aimed at directly testing the association between domain-general reasoning abilities and gambling cognitions, in two samples of (a) individuals from the community who present a detectable level of gambling but do not present gambling problems (henceforth, individuals with non-problematic gambling, NPG), and (b) treatment-seeking patients with gambling disorder (PGD).

Reasoning abilities (i.e., the independent variables in our study) were assessed using the matrices task of the WAIS-IV intelligence scale (Wechsler, 2008), and the Berlin Numeracy Test [BNT (Cokely et al., 2012)], for abstract and probabilistic reasoning, respectively, mirroring the two mechanisms described earlier. These two measures have good validity and reliability. The BNT is a sound index of probabilistic reasoning in practice (Cokely et al., 2018), namely individuals’ easiness to deal with basic probabilistic operations from real-life problems (Lipkus and Peters, 2009; Cokely et al., 2012). The matrix reasoning subtest of the WAIS-IV assesses non-verbal perceptual reasoning abilities, and is considered to be a reliable measure of fluid intelligence (Bugg et al., 2006; Wechsler, 2008; Stephenson and Halpern, 2013; Gignac, 2014; Green et al., 2017; Kim and Park, 2018). This mostly overlaps with the g-factor (Spearman, 1927; Tranel et al., 2008; Jaeggi et al., 2010).

On the side of dependent measures, gambling-related cognitions were assessed using the GRCS, described earlier. Complementarily, severity of potentially disordered gambling was assessed with the South Oaks Gambling Screen [SOGS, Spanish version (Echeburúa et al., 1994)].

In view of the evidence briefly reviewed earlier, we expect participants in the PGD sample to present a small-to-moderate disadvantage in the matrices and BNT tests, and much stronger dysfunctional/distorted gambling-related cognitions, relative to participants in the NGD sample. Yet, our main hypotheses, specifically regarding the relationships between BNT/matrices scores and gambling-related cognitions, remain open. Firstly, across samples, we will estimate the independent contribution of domain-general reasoning scores to the five domains of gambling-related cognition. Secondly, associations (or their absence) between reasoning and gambling-related cognitions will be tested in the two samples separately. Support for the existence (H_1_) or inexistence (H_0_) of such links will be assessed using Bayes factors.

## Materials and Methods

### Participants

The study sample comprised 135 participants, divided in 77 treatment-seeking patients with gambling disorder (PGD) and 58 participants with non-problematic gambling involvement (NPG). Characteristics of the two samples are reported in Table 2. Participants in the PGD group had a diagnosis of gambling disorder, as established by their therapist based on DSM5 criteria, and they had abstained from gambling for 15 days or more. The NPG group consisted of individuals with different degrees of involvement in gambling activities (with the minimum being “having ever gambled”). A specific exclusion criterion for NPG was presenting a gambling pattern severe enough to be classified as a disordered gambler [i.e., ≥5 in SOGS; (Stinchfield, 2002)]. The rest of exclusion criteria were similar for both groups, i.e., having ever been diagnosed or treated for any psychopathology (beyond gambling disorder in the case of PGD), and any history of neurological disease or brain trauma causing unconsciousness for 10 min or longer. Common exclusion criteria were assessed with a semi-structured interview.

**TABLE 2:** Descriptive statistics of all measured variables, and Bayes factors (based on the non-parametric Mann-Whitney statistic for quantitative variables and a Bayesian contingency table test for gender) and *p*-values (Welch’s *t*-tests for quantitative variables, and χ^2^-test for gender) for the differences between samples (patients with gambling disorder vs. individuals with non-problematic gambling).

	*Sample*	*Mean*	*SD*	*Min.*	*Max.*	*BF*_10_	*p*
*Gender*	*PGD*	2F/75M				0.60	0.733
	*NGD*	1F/57M					
*Age*	*PGD*	36.18	11.42	19	61	0.29	0.142
	*NGD*	33.62	8.75	20	61		
*Education ys.*	*PGD*	12.34	3.92	5	24	0.95	0.064
	*NGD*	13.48	3.19	7	20		
*Matrices*	*PGD*	97.08	16.31	65	130	3.79	0.008
	*NGD*	104.31	14.61	75	140		
*BNT*	*PGD*	0.82	0.96	0	3	1.64	0.011
	*NGD*	1.26	1.00	0	3		
*Expectancy*	*PGD*	3.95	1.68	1	7	>100	<0.001
	*NGD*	1.49	0.71	1	4		
*Inability to stop*	*PGD*	4.26	1.66	1	7	>100	<0.001
	*NGD*	1.19	0.51	1	4		
*Control illusion*	*PGD*	2.59	1.40	1	7	>100	<0.001
	*NGD*	1.25	0.52	1	4		
*Predictive control*	*PGD*	3.75	1.53	1	7	>100	<0.001
	*NGD*	1.48	0.64	1	4		
*Interpretative bias*	*PGD*	4.75	1.79	1	7	>100	<0.001
	*NGD*	1.50	0.86	1	5		
*SOGS*	*PGD*	10.35	2.99	3	17	>100	<0.001
	*NGD*	0.62	0.93	0	3		

### Procedure

Patients with gambling disorder were recruited from different associations of rehabilitated gamblers in Andalucía (Spain), whereas NPG were recruited using convenience and snowball sampling methods among researchers’ and patients’ acquaintances, and using advertisements.

All participants were recruited across different phases of a more ambitious multi-stage research project (GBrain, and GBrain-2, see section “Funding”), with the different stages having slightly different aims and assessment protocols (with some measures being common to all phases and others present in only some of them). The participants included in the present study were the ones from all the phases of the project that were assessed with both the Matrices test for abstract reasoning, and the BNT for probabilistic reasoning (i.e., the two main independent variables involved in the hypotheses articulated earlier).

Across phases, PGD and NPG participants were sampled from similar social milieus, and groups were intendedly matched in sociodemographics, including gender, age and education years (but not psychological/cognitive characteristics; please see complementary information about matching in the section “Preliminary Analyses”).

In all phases, the protocol consisted of a set of questionnaires and neuropsychological tasks, administered in a quasi-randomized order, in a single session that lasted approximately 2 h. Some participants were invited to participate in an extra session in a different day, in which psychophysiological or neuroimaging measures were recorded. There is thus some overlap between the current sample and the one in other studies of our research group: Megías et al. (2018), 33.3%; Navas et al. (2016, 2017b), 60%; Perales et al. (2017), 47.4%; Perandrés-Gómez et al. (2020), 97%; Ruiz de Lara et al. (2018), 34.1%; and Navas et al. (2017a), 52.6%.

Participants were debriefed about study aims and signed an informed consent prior to their participation, and received a €10/hour compensation. In the case of patients, the compensation was paid via an authorized relative. The study was approved by the Ethic Committee of the University of Granada and complied with the Helsinki Declaration.

### Instruments

#### Matrix Reasoning Task [WAIS-IV (Wechsler, 2008)]

This instrument consists of 26 sequences of geometric figures, with each one following a unique organizational pattern, and a blank cell. Participants are asked to guess the underlying logic in the sequence, and to fill the blank cell with the option that best fits among the five possible alternatives. This is a standardized task that has excellent psychometric properties and is adapted for Spanish populations (Wechsler, 2012).

#### Berlin Numeracy Test [BNT (Cokely et al., 2012)]

This is a paper-and-pencil test in which participants are asked to answer 4 different questions on probability in ascending order of difficulty [e.g., *Imagine we are throwing a five-sided die 50 times. On average, out of these 50 throws, how many times would this five-sided die show an odd number (1, 3 or 5*)?]. A final score of numeracy skills is calculated as the sum of correct answers.

#### Gambling-Related Cognitions Scale [GRCS; Raylu and Oei, 2004; Spanish version: Del Prete et al., 2017)]

This is a self-reported measure of gambling-related cognition based on Raylu and Oei′s model. It consists of 23 items to be answered using a five-point Likert scale that assess five cognitive distortions: inability to stop gambling (e.g., *My desire to gamble is so overpowering*), gambling expectancies (e.g., *Gambling makes things seem better*), predictive control (e.g., *Losses when gambling, are bound to be followed by a series of wins*), illusion of control (e.g., *I have specific rituals and behaviors that increase my chances of winning*), and interpretative bias (e.g., *Relating my winnings to my skill and ability makes me continue gambling*). Given that individuals in the PGD group had been in therapy for some time (from 15 days to 6 months), these participants were specifically instructed to refer their answers to the GRCS items to the time when they initiated treatment [see also (Navas et al., 2017a)].

This scale has shown good psychometric properties (Del Prete et al., 2017). In the present study, internal consistency values (Cronbach’s α) were 0.866, 0.914, 0.709, 0.826, and 0.920 for gambling expectancies, inability to stop, illusion of control, predictive control and interpretive bias, respectively, and 0.963 for the total scale.

#### South Oaks Gambling Screen [SOGS (Lesieur and Blume, 1987); Spanish Version (Echeburúa et al., 1994)]

This instrument was used to assess disordered gambling symptoms’ severity. The Spanish version has shown good psychometric properties. For this study, SOGS showed an excellent level of internal consistency (Cronbach’s α = 0.929).

### Statistical Analyses

Descriptive statistics are provided for age, education years, gender composition, WAIS-IV matrices scores, BNT scores, SOGS total severity scores, and the five dimensions of the GRCS questionnaire (gambling expectancies, inability to stop, control illusion, predictive control, and interpretative bias). For quantitative or quasi-quantitative variables, these descriptives include mean, standard deviation, and maximum and minimum values. These descriptives are complemented with Bayesian and frequentist tests to check for differences between participants showing non-problematic gambling involvement (NPG) and patients with gambling disorder (PGD). Scores in the five dimensions of the GRCS are submitted to a first multivariate analysis of covariance (MANCOVA), with group (sample: PGD, NPG) as a between-participant factor, and WAIS-IV matrices score as a continuous predictor. These are followed by GRCS dimension-by-dimension analyses of covariance (ANCOVA), with the same independent variables. The same analyses will be performed with BNT (instead of matrices) scores as continuous predictor.

Given the nature of the dependent variables involved, these analyses are likely to be affected by two limitations: (a) violation of homogeneity of covariance matrices and multivariate normality assumptions, and (b) the unsuitability of null-hypothesis significance testing (NHST) to provide evidence in favor of the null hypothesis. In view of that, non-parametric correlations (Kendall’s τ) will be computed for correlations of each GRCS subscore with matrices and BNT scores. These correlations will be interpreted using bidirectional Bayes factors (BF_10_) instead of NHST.

Regarding these statistical analyses, there are two important points that require further consideration. First, we did not use stratified sampling (or any other method to ensure populational representativity; see section “Limitations and Final Remarks”), but the sampling strategy and the inclusion/exclusion criteria were very similar for the two groups, and we did not force matching on psychological/cognitive variables [please see Perandrés-Gómez et al. (2020), for a discussion on the consequences of IQ non-matching in cross-sectional analyses of a sample largely overlapping with the present one]. Using convenience samples of gamblers with and without gambling problems is quite a standard practice in correlational research in the field (Barrada et al., 2019). Still, and in order to surpass the problems that this sample composition may cause, we ran analyses with the whole sample, while controlling for group (first part of the section “Main Analyses”), with the whole sample without controlling for group (Supplementary Materials), and with the two groups separately (second part of the section “Main Analyses”). As detailed below, results were robust across statistical approaches.

And second, please note that frequentist tests are aimed at checking for statistical significance of effects (i.e., whether the observed test statistic is extreme enough for the null hypothesis to be rejected), so null results can be explained as resulting from either the absence of an effect or the lack of power of the test. That implies that frequentist tests cannot distinguish between evidence of absence and absence of evidence (Altman and Bland, 1995). In the present study, however, we are as much interested on the possible inexistence of certain relations as we are in their existence. Bayesian tests expressed in the form of Bayes factors (BF_10_) are aimed at comparing two models of the world, one in which the effect of interest is zero, and another one in which it is non-zero (with a given probability density distribution over the populational effect size). These two models representing the null and the alternative hypothesis are treated symmetrically, in such a way that BF_10_ < 1 is interpreted as supporting the null, whereas BF_10_ > 1 is interpreted as supporting the alternative. The arbitrary thresholds to consider evidence in favor of one or the other substantial enough vary across reference guidelines, so BFs will be interpreted here as strictly continuous measures of evidence (Dienes, 2014). For a discussion on equivalence tests and Bayes factors as tools to establish evidence for the null, see Lakens et al. (2020).

Data and reproducible analysis files are fully available in the OSF framework^1^.

## Results

### Preliminary Analyses

Table 2 shows group means, maximum, and minimum values, and standard deviations for age, education years, matrices, BNT, SOGS severity, and GRCS dimensions scores; proportions for gender; as well as Bayes factors and *p*-values for differences between groups in all variables. Detailed distributions for all these variables across groups are reported in the Supplementary Materials.

As expected, the two groups differed in SOGS and GRCS scores, and were closely matched in gender composition and mean age. Although education years was also controlled across phases of the project, the pooling of samples across phases made the difference between groups in this variable to get close to the significance threshold (*p* = 0.064), and to yield a virtually uninformative BF (BF_10_ ≈1).

The two groups, however, differed in both Matrices and BNT scores. In other words, differences in reasoning abilities remained in spite of control of sociodemographic variables. Actually, a MANCOVA with BNT and matrices scores as dependent variables, group as independent variable, and sociodemographics (age, gender, and education years) as covariates yielded significant effects for both the multivariate effect (Wilks’ λ = 0.910, *p* = 0.002), and the univariate effects [F (1, 130) = 8.109, *p* = 0.005; and F (1, 130) = 8.335, *p* = 0.005, for matrices and BNT scores, respectively]. In other words, despite sociodemographic matching, general reasoning scores remained associated with GD, which is in line with the abovementioned evidence of links between reasoning abilities and risk of being diagnosed with GD.

### Main Analyses

The MANCOVA with group as between-participants factor, matrices score as continuous predictor, and GRCS subscores as dependent variables, yielded a significant effect for group, Wilks’ λ = 0.378, F (5, 128) = 42.181, *p* = 0.001, but not for the matrices score, Wilks’ λ = 0.991, F (5, 128) = 0.231, *p* = 0.948. Table 3 (left panel) shows the results of separate ANCOVAs for the five GRCS dimensions. In accordance with the global MANCOVA, all dependent variables showed significant effects of group, but not of matrices score.

**TABLE 3:** Results of separate ANCOVAs for GRCS dimensions as dependent variables, and Group and continuous predictors (left: WAIS matrices, right: BNT) as independent variables.

		*WAIS matrices*			*BNT*		
*IV*	*DV*	*MSE*	*F* (1, 132)	*P*	*MSE*	*F* (1, 132)	*p*
*Group*	*EXP*	1.836	108.850	<0.001	1.812	110.305	<0.001
	*IS*	1.707	182.762	<0.001	1.691	184.538	<0.001
	*CI*	1.245	47.697	<0.001	1.239	47.950	<0.001
	*PC*	1.517	112.572	<0.001	1.506	113.375	<0.001
	*IB*	2.160	161.027	<0.001	2.119	164.100	<0.001
*Covariate*	*EXP*	1.836	0.234	0.629	1.812	2.001	0.160
*(Matrices/BNT)*	*IS*	1.707	0.006	0.938	1.691	1.289	0.258
	*CI*	1.245	0.072	0.789	1.239	0.772	0.381
	*PC*	1.517	0.085	0.772	1.506	1.027	0.313
	*IB*	2.160	0.124	0.725	2.119	2.645	0.106

*IV, Independent variable; DV, Dependent Variable; EXP, Gambling Expectancies, IS, Inability to Stop, CI, Control Illusion, PC, Predictive Control, IB, Interpretative Bias, BNT, Berlin Numeracy Test.*

Similarly, the MANCOVA with group as between-participants factor, BNT score as continuous covariate, and GRCS subscores as dependent variables yielded a significant effect for group, Wilks’ λ = 0.374, F (5, 128) = 42.884, *p* < 0.001, but not for the BNT score, Wilks’ λ = 0.977, F (5, 128) = 0.607, *p* = 0.695. Table 3 (right panel) shows the results of separate ANCOVAs for the five GRCS dimensions. In accordance with the global MANCOVA, all dependent variables showed significant effects of group, but not of BNT score^2^.

**FIGURE 1:**
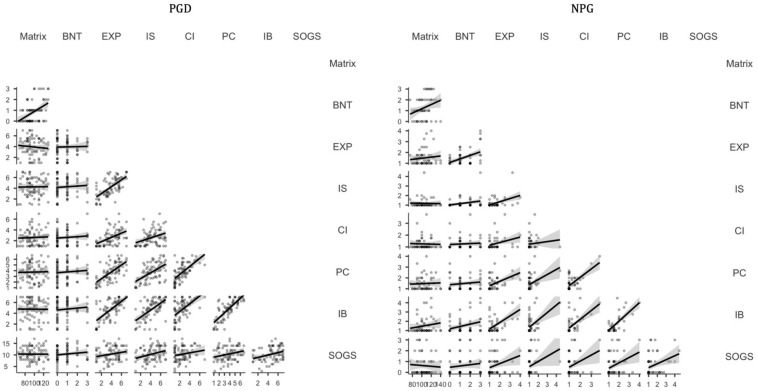
Graphic depiction of the correlation matrix for all variables of interest across groups (PGD, patients with gambling disorder; NPG, Individuals in non-problematic gambling; Matrix, WAIS matrices scores; BNT, Berlin Numeracy Test; EXP, Gambling Expectancies; IS, Inability to Stop; CI, Control Illusion; PC, Predictive Control; IB, Interpretative Bias; SOGS, Gambling severity).

The Box’s test [χ^2^ (15) = 201, *p* < 0.001], and the Shapiro-Wilks’ test [*W* = 0.875, *p* < 0.001], showed clear violations of the homogeneity of covariance matrices and multivariate normality assumptions, respectively. In view of that, we computed non-parametric correlations (Kendall’s τ) between reasoning abilities and GRCS dimensions for the two groups separately, and interpreted the evidence portrayed by them using bidirectional Bayes factors (BF_10_), computed with the default settings in JASP software (JASP Team, 2019). Figure 1 and Table 4 show the results of these analyses for the PGD and the NPG group, respectively.

**TABLE 4:** Bayesian correlation tests (bidirectional Bayes factors for Kendall’s τ) between variables of interests in PGD and NPG samples.

		*Age*	*Education*	*Matrices*	*BNT*	*EXP*	*IS*	*CI*	*PC*	*IB*
**PGD**										
*Education*	τ	–0.31								
	BF_10_	>100								
*Matrices*		–0.07	0.40							
		0.22	>100							
*BNT*		–0.27	0.41	0.34						
		48.47	>100	>100						
*EXP*		–0.03	–0.04	–0.08	–0.02					
		0.16	0.18	0.27	0.15					
*IS*		0.00	–0.09	–0.02	0.07	0.46				
		0.15	0.28	0.16	0.22	>100				
*CI*		–0.08	–0.04	0.00	0.05	0.38	0.27			
		0.24	0.16	0.15	0.19	>100	67.17			
*PC*		–0.14	0.05	0.00	0.07	0.49	0.40	0.51		
		0.71	0.18	0.15	0.22	>100	>100	>100		
*IB*		–0.10	0.00	–0.02	0.05	0.50	0.47	0.42	0.61	
		0.35	0.15	0.16	0.18	>100	>100	>100	>100	
*SOGS*		–0.06	–0.05	0.00	0.11	0.13	0.22	0.11	0.18	0.22
		0.20	0.19	0.15	0.38	0.53	7.59	0.42	1.93	6.83
**NPG**										
*Education*	τ	–0.18								
	BF_10_	1.20								
*Matrices*		0.23	0.11							
		4.24	0.36							
*BNT*		0.05	0.29	0.25						
		0.19	26.54	7.42						
*EXP*		–0.10	0.05	0.05	0.27					
		0.30	0.19	0.20	15.04					
*IS*		0.03	0.03	–0.04	0.22	0.35				
		0.18	0.18	0.19	3.42	>100				
*CI*		0.03	–0.04	–0.02	0.12	0.33	0.32			
		0.18	0.19	0.18	0.39	>100	76.20			
*PC*		–0.16	0.07	0.02	0.13	0.44	0.38	0.40		
		0.82	0.23	0.17	0.49	>100	>100	>100		
*IB*		–0.13	0.08	0.11	0.19	0.42	0.37	0.55	0.62	
		0.48	0.24	0.35	1.64	>100	>100	>100	>100	
*SOGS*		–0.02	–0.15	–0.03	0.16	0.36	0.37	0.21	0.30	0.32
		0.17	0.69	0.18	0.76	>100	>100	2.36	45.14	81.42

*EXP, Gambling Expectancies; IS, Inability to Stop; CI, Control Illusion; PC, Predictive Control; IB, Interpretative Bias; BNT, Berlin Numeracy Test; SOGS, Gambling symptoms’ severity.*

As expected, in both groups, substantial correlations were found between the different subdimensions of GRCS. In the NPG group, the SOGS score correlated positively with all GRCS dimensions, with the strength of evidence for H_1_ ranging from BF_10_ = 2.36 to BF_10_ > 100. Correlations between SOGS and GRCS were weaker in the PGD group, with only three BFs above 1, i.e., for inability to stop (BF_10_ = 7.59), interpretative bias (BF_10_ = 6.83), and predictive control (BF_10_ = 1.93, anecdotal)^3^. BNT and matrices also correlated positively between them, and with education years, and negatively with age.

Most importantly, BFs for correlation coefficients between reasoning abilities (matrices and BNT) and GRCS scores mostly provided moderate (BF_10_ < 0.33) evidence in favor of the null hypothesis. The only exceptions (i.e., BF_10_ > 1) were the BF_10_ = 15.04, Kendall’s τ = 0.27 between BNT and gambling expectancies, the BF_10_ = 3.41, Kendall’s τ = 0.22 between BNT and inability to stop, and the BF_10_ = 1.64 (anecdotal), Kendall’s τ = 0.19 between BNT and interpretative bias, in the NPG group. In other words, there is some weak evidence of a direct link between BNT and some gambling-related cognitions (mainly excluding gambling biases) in the NPG group, with stronger cognitions in individuals with higher BNT scores. There were not any cases in which evidence supported an inverse relationship between reasoning abilities and gambling-related cognitions.

## Discussion

The goal of the present study was to explore the relationships between domain-general reasoning abilities and gambling-related cognitions in non-problematic gamblers (NPG) and patients with gambling disorder (PGD). Results from NHST (MANCOVAs on the association between BNT/Matrices and gambling-related cognitions, and subsequent dimension-by-dimension ANCOVAs) did not yield any significant associations. This result holds regardless of whether group (PGD, NPG) was included in the model or not. Subsequent Bayesian analyses yielded consistent support for the null hypothesis, i.e., no association between BNT/Matrices and gambling-related cognitions, except for anecdotal-to-substantial support for positive associations in the NPG subsample between BNT, on the one side, and gambling expectancies, inability to stop, and interpretative bias, on the other.

These results converge with the ones of some previous works. For instance, Perales et al. (2017) found gamblers with stronger biases to perform better in a causal learning task than those with weaker biases. This result was interpreted as originating in the fact that gambling-related cognitive distortions are significantly more intense in gamblers preferring skill-based games (i.e., sports betting, casino and card games) than in those preferring chance games (i.e., slots, bingo, or lottery) [see also (Myrseth et al., 2010; Navas et al., 2017b; Mallorquí-Bagué et al., 2019)]. Individuals preferring skill-based games are, on average, younger, better educated, and more sensitive to reward (Navas et al., 2017b), so that their distorted beliefs about gambling are unlikely to be originated in any general-domain reasoning disadvantage. Relatedly, Xue et al. (2012a) found students with higher cognitive abilities (intelligence and executive function) to be more prone to show the gambler’s fallacy. And in Lambos and Delfabbro (2007) disordered gamblers were found to be more susceptible to cognitive biases than non-gamblers and non-disordered gamblers, but no significant differences were observed between the three groups for their knowledge of gambling odds [see also (Benhsain et al., 2004)].

This lack of substantial inverse relationships between domain-general reasoning abilities and gambling-related cognitions renders two theoretical puzzles unresolved. First, to describe the mechanisms responsible for bias generation and their activation during and between gambling sessions; and, second, accounting for the seemingly robust link between domain-general cognitive abilities and the risk developing gambling problems, *without* the mediation of gambling-related distorted cognitions.

With regard to the first question, a possible solution arises from the cognitive switching (Sévigny and Ladouceur, 2003) hypothesis. According to this hypothesis, individuals with disordered gambling “switch off” their rational beliefs during gambling, so that their behavior becomes governed by features of the game or the gambling device, and “switch them on” again when they finish. In other words, in-game behavior and cognitions remain impermeable to general-domain reasoning.

The cognitive switching hypothesis is inspired by dual-process models of cognition, according to which two competing systems, the intuitive and the analytic, filter the information necessary to control action. The intuitive system is regarded as fast, efficient, and heuristic-based, whereas the analytic system is slower and more effortful, but also more rational (Armstrong et al., 2020). The term *cognitive reflection* has been coined to denote the degree to which an individual is more or less willing to invest the necessary cognitive resources to engage in analytic thinking [see (Stange et al., 2018), for a discussion of its potential link with gambling]. Importantly, being less prone to cognitive reflection, especially under certain environmental and affective circumstances, does not imply having poorer reasoning abilities, but somehow eschewing the effort to use them, especially when motivated to do so. In words of Armstrong et al. (2019), “gamblers are often unlikely or unwilling to reflect on the veracity of beliefs as they are often used to justify gambling behaviors” (p. 183) [see also (Emond and Marmurek, 2010; Armstrong et al., 2019; Cosenza et al., 2019)]. This mechanism reminds of the “tilt” phenomenon in poker (Barrault et al., 2014), and some recent studies using functional magnetic resonance imaging (fMRI) (Xue et al., 2011), and transcranial direct current stimulation (tDCS) (Xue et al., 2012b) also indirectly support it.

A second, non-exclusive possibility is that some gamblers do remain reflective during gambling episodes, but they invest their cognitive resources in trying to “outsmart” the gambling device, and to find causal patterns where there are not any. Indirect evidence supporting this mechanism comes from the abovementioned reports that, especially in some sociodemographic sectors, individuals with preserved –or even superior– cognitive skills are more vulnerable to certain gambling-related fallacies. To our knowledge, there is no direct evidence of this mechanism, although the deleterious effects of trying to outsmart random devices on judgment and decision-making are well known [see (Gaissmaier and Schooler, 2008)].

That connects with a third possibility, emerging from the putative interaction of domain-general reasoning skills with age and/or education. Actually, when matrices scores were allowed to interact with age and education years (see Supplementary Materials, second section), some non-significant trends suggested that, in younger and more educated individuals, matrices scores were positively associated with GRCS scores, whereas in older and less educated individuals the association was non-existing or in the opposite direction. It is definitely premature to make any inferences from these trends, but they open the possibility that in younger, more educated people, distorted gambling cognitions were fueled by domain-general reasoning skills, whereas in older, less educated gamblers, poorer reasoning skills were a risk factor for developing gambling-related biases. Additionally, this interaction would explain why some studies have found no associations whatsoever between reasoning skills and gambling-related biases, whereas others have found a direct link (Xue et al., 2012a; Perales et al., 2017).

In summary, low domain-general reasoning skills are not necessary to develop gambling-related distorted beliefs, which reinforces the idea that, at least in some gamblers, in- or about-game emotion-laden states (e.g., urges triggered by conditioned cues, or negative affect caused by losses) can take control over gambling-related cognition, and probably motivate the individual to stick to irrational cognitions. Such possibility is one of the main tenets of the GSM, according to which the main source of gambling-related cognitive distortions is motivated reasoning, that is, the individual’s tendency to regulate affect by overestimating their degree of control or reinterpreting gambling outcomes in a more favorable, ego-protecting light (Navas et al., 2017b, 2019; Ruiz de Lara et al., 2019). Whether this motivated reasoning mechanism is specific to some gamblers (more educated, younger ones) or generalizes to a wider range of individuals remains an open question for future research.

The second puzzle, namely the moderate but seemingly robust relationship of intelligence and abstract reasoning with gambling problems without the mediation of gambling related cognitions, seems more difficult to address. In our sample, this link held for GD diagnosis across groups, but not for severity of gambling problems within groups, and its interpretation is limited by features of the design. This result resonates with the one from Rai et al. (2014), in which a link between IQ and gambling problems was also corroborated at the populational level, but no association was found between IQ and non-problematic gambling. Unfortunately, none of the possible explanations for this link has been explored in detail. Tentatively, the association between poorer reasoning abilities and a higher risk of developing gambling problems can be partially accounted for by the overlap between these abilities and aspects of executive function as self-control and top-down regulation of impulses (Meldrum et al., 2017). A detailed review of the role of executive functions related to cognitive control in gambling problems, and its neurobiological correlates, can be found at Moccia et al. (2017).

Clinical implications of our results, and the abovementioned related ones, are far-reaching. Gambling-related cognitions are hard to restructure, and the efficacy of cognitive therapy, although well-established, remains modest (Petry et al., 2017). Furthermore, individuals with problematic gambling are normally reluctant to change their beliefs when faced with disconfirming evidence, and often counterargument it (Delfabbro et al., 2006). In a variety of domains, this sort of reluctance has been related to the fact that, when motivated to maintain a given belief, individuals perceive information disconfirming it as confronting or uncomfortable (Gilbert et al., 1990; Mezirow, 1990; Stange et al., 2018). In consequence, altering beliefs will not only require more (or more accurate) information, but an increased degree of metacognition about how motives to gamble and to regulate emotions derived from gambling (and its consequences) relate to one’s beliefs (Wells, 2009; Lindberg et al., 2014b; Caselli and Spada, 2016).

### Limitations and Final Remarks

Results of our study should also be understood considering at least five main limitations. First, we cannot establish causal associations between the variables examined, since this is a cross-sectional study. Second, since the majority of the participants are male, generalizability to the entire population of gamblers should not be taken for granted. Third, assessing psychological constructs using self-report questionnaires may not fully represent the cognitive processes involved, and social desirability effects are possible. Fourth, no power analysis was performed *a priori* to determine sample size. As noted earlier, participants in this study were the ones in a larger project who had been assessed with all the measurements of current interest. This problem is, however, partially palliated by the use of Bayes factors, that provide evidence in support of the null or the alternative hypothesis in a continuous fashion, so that no dichotomous decisions leading to type I or type II errors are made. And fifth, we did not use stratified sampling (or any other method to ensure populational representativity), which means that the sampling strategy and the inclusion/exclusion criteria were very similar for the two groups, and we did not force matching on psychological/cognitive variables. That implies that the proportion of PGD in our sample is much larger than in the general population, but there are no reasons to expect substantial alterations of the correlations between psychological variables. Given that there is an association between gambling problems, on the one hand, and both stronger gambling-related biases and lower reasoning skills, on the other, the overrepresentation of PGD could have artificially inflated correlations between the latter when group was not controlled for (supplementary analyses). Despite this risk of inflation, gambling-related cognitions and domain-general reasoning remained mostly disconnected.

On the side of strengths, although some previous studies had explored the relationship between reasoning abilities and gambling-related beliefs, to our knowledge, this is the first one simultaneously assessing two core constructs of domain-general reasoning directly relevant to gambling (abstract and probabilistic reasoning), and their relationship with different dimensions of gambling-related cognitions in individuals without problem gambling and patients with gambling disorder. Additionally, the inclusion of Bayesian analyses allows to symmetrically assess the evidential support in favor of the null or the alternative hypothesis. Our results evidence that probabilistic and abstract reasoning abilities are mostly unrelated to the intensity of distorted gambling-related beliefs, and are thus unlikely to protect gamblers from them. This pattern or results reinforces the idea that distorted cognitions do not originate in a general lack of understanding of probability or low fluid intelligence, but probably result from motivated reasoning.

## Data Availability Statement

The datasets presented in this study can be found in the OSF repository: https://osf.io/8ksxa/.

## Ethics Statement

The studies involving human participants were reviewed and approved by Ethic Committee of the University of Granada. The patients/participants provided their written informed consent to participate in this study.

## Author Contributions

JP, JN, and IM: conceptualization and writing–review and editing. JP: formal analysis. JP and JN: methodology. IM and JN: writing – original draft. All authors contributed to the article and approved the submitted version.

## Conflict of Interest

JP was an editor for the Special Topic in which this article is included. This article was however handled by an independent editor, unknown to any of the authors, and different from the other editors of the Topic. The remaining authors declare that the research was conducted in the absence of any commercial or financial relationships that could be construed as a potential conflict of interest.

## Footnotes

1 https://osf.io/8ksxa/

2 These MANCOVAs were re-run including either education years or age as covariates, and allowing both covariates to interact with either BNT or Matrices (i.e., estimating the possible dependence of the effect of reasoning abilities on age or education years). Results from those analyses are reported in the second section, Supplementary Table 1 and Supplementary Figure 2. The potential relevance of these results is detailed in the section “Discussion.”

3 The different size of correlations in the two groups could be due to range restriction in the SOGS scale. As shown in Supplementary Figure 1, GRCS scores showed a large range of variation in the PGD group, but SOGS scores in that group were more tightly concentrated around the mean (*M* = 10.35, *SD* = 2.99), which results from the fact that PGD in our study were severe enough to have sought treatment. Still, results in this group are also consistent with the fact that inability to stop and interpretative biases are the best GRCS indicators of severity at pathological levels (Michalczuk et al., 2011; Del Prete et al., 2017).
