# Supplementary material for: Gambling-Specific Cognitions Are Not Associated With Either Abstract or Probabilistic Reasoning: A Dual Frequentist-Bayesian Analysis of Individuals With and Without Gambling Disorder
Source: Front Psychol. 2021 Jan 26;11:611784. doi: 10.3389/fpsyg.2020.611784 (PMC7873942; doi:10.3389/fpsyg.2020.611784)

**Supplementary materials**

**Distribution of scores for all measurements of interests across groups**

Figure A1 displays violin plots for distributions of age, education years, matrices scores, GRCS subscores, and SOGS severity scores, and frequency histogram for the number of correct responses in the BNT, across groups. Contrast statistics and BFs for the observed differences are reported in the main manuscript.

**BNT and matrices effects controlling for age and education**

The MANCOVAs described in the main analyses were re-run including either education or age as covariates, and allowing both covariates to interact with either BNT or Matrices (i.e. estimating the possible dependence of the effect of reasoning abilities on GRCS scores on age or education years). Table A1 shows the results for the four tested models.

In terms of significance, results were virtually identical to the ones described in the main text, with the addition of a significant multivariate effect of education on GRCS scores in the third model in Table A1. However, a number of other effects were close to significance, including the ones of matrices, matrices x age, and matrices x education. Marginal significance and the multiplicity of tests yield these effects virtually non-interpretable. Still, in order to visualize them, total predicted GRCS scores (the sum of the four GRCS dimensions) are displayed in Figure A2. In general, the relationship between education and biases trend in the positive direction, with younger individuals showing this pattern more clearly, and older ones showing a weaker one, or even a trend in the opposite (negative) direction. The potential relevance of these trends is presented in the discussion section of the main manuscript.

**Full-sample analysis**

In view of the tight association between disordered gambling symptoms’ severity (SOGS scores) and the strength of gambling-related cognitions (GRCS), it could be argued that including group (PGD vs NPG) in main analyses could overshadow the impact of reasoning abilities on gambling-specific beliefs and biases.

Disregarding group when analyzing the putative effect of reasoning abilities would increase the likelihood that other variables correlating with group (i.e. any variables the two groups differ at, due to manipulation by selection) got confounded with reasoning abilities, artificially boosting the correlation between them and GRCS scores. Yet, for the sake of transparency and robustness, we have re-run the cross-sectional MANCOVAs described in the main text, but with reasoning abilities as the only independent variables (thus disregarding group), and GRCS scores as dependent variables, followed, by dimension-by-dimension Bayesian correlational analyses.

Despite the large sample size, neither the multivariate effect of matrices nor the one of BNT on GRCS scores were significant [Wilks’ λ = 0.965, F (5, 129) = 0.949, p = 0.452; and Wilks’ λ = 0.985, F (5, 129) = 0.402, p = 0.847].

Bayesian correlations did not change main conclusions either. As expected, increasing the range of severity (and given the large differences between groups in GRCS scores) also increased the strength of the correlations between SOGS and GRCS scores. Most importantly, although the sign of correlations between reasoning abilities and GRCS changed from positive to negative (relative to separate group analyses shown in the main text; see Table A2 and Figure A3), these correlations remained close to zero, and Bayes factors systematically supported the null hypothesis (except for the BF_10_ = 1.713 anecdotally supporting the correlation between matrices score and inability to stop).

Complementarily to these correlational whole-sample analyses, we also carried out Bayesian regression analyses for each one of the GRCS dimensions, with age and education years as covariates, and either BNT or matrices scores as main predictors (in all cases, the BF_10_ was computed pitching a null model including age and education years, against a comparison model that included either BNT or matrices upon the null model; BNT and matrices were never included in the same model to avoid competition between them). BF_10_ values were 1.288 and 0.331 for the effects of matrices and BNT scores on gambling expectancies, 0.706 and 0.324 on inability to stop, 0.344 and 0.332 on control illusion, 0.650 and 0.430 on predictive control, and 0.674 and 0.356. In other words, controlling for age and education years left results qualitatively unaltered.

**Table A1.** Results of separate MANCOVAs for multivariate effects of BNT and matrices effects on GRCS, with either age an education as covariates.

| ***Model*** | ***Effect*** | ***Pillai's trace*** | ***F*** | ***p*** |
| --- | --- | --- | --- | --- |
| **BNT controlling for age** | |  |  |  |
|  | *BNT* | 0.030 | 0.773 | 0.571 |
|  | *Age* | 0.079 | 2.158 | 0.063 |
|  | *Group* | 0.627 | 42.338 | *< 0.001* |
|  | *BNT x Age* | 0.047 | 1.243 | 0.293 |
|  |  |  |  |  |
| **BNT controlling for education** | |  |  |  |
|  | *BNT* | 0.030 | 0.793 | 0.557 |
|  | *Education* | 0.096 | 2.685 | *0.024* |
|  | *Group* | 0.625 | 42.058 | *< 0.001* |
|  | *BNT x Education* | 0.055 | 1.467 | 0.205 |
|  |  |  |  |  |
| **Matrices controlling for age** | |  |  |  |
|  | *Matrices* | 0.077 | 2.097 | 0.070 |
|  | *Age* | 0.078 | 2.135 | 0.065 |
|  | *Group* | 0.621 | 41.255 | *< 0.001* |
|  | *Matrices x Age* | 0.072 | 1.941 | 0.092 |
|  |  |  |  |  |
| **Matrices controlling for education** | |  |  |  |
|  | *Matrices* | 0.075 | 2.042 | 0.077 |
|  | *Education* | 0.079 | 2.168 | 0.062 |
|  | *Group* | 0.609 | 39.322 | *< 0.001* |
|  | *Matrices x Education* | 0.074 | 2.005 | 0.082 |

**Table A2.** Bayesian correlation tests (bidirectional Bayes factors for Kendall’s t) between for variables of interests in the full sample.

|  |  | ***Age*** | ***Education*** | ***Matrices*** | ***BNT*** | ***EXP*** | ***IS*** | ***CI*** | ***PC*** | ***IB*** |
| --- | --- | --- | --- | --- | --- | --- | --- | --- | --- | --- |
| ***Education*** | τ | -0.263 |  |  |  |  |  |  |  |  |
|  | BF₁₀ | > 100 |  |  |  |  |  |  |  |  |
| ***Matrices*** |  | 0.026 | 0.308 |  |  |  |  |  |  |  |
|  |  | 0.124 | > 100 |  |  |  |  |  |  |  |
| ***BNT*** |  | -0.171 | 0.385 | 0.32 |  |  |  |  |  |  |
|  |  | 8.341 | > 100 | > 100 |  |  |  |  |  |  |
| ***EXP*** |  | -0.026 | -0.08 | -0.121 | -0.04 |  |  |  |  |  |
|  |  | 0.125 | 0.289 | 0.956 | 0.142 |  |  |  |  |  |
| ***IS*** |  | 0.049 | -0.126 | -0.136 | -0.068 | 0.634 |  |  |  |  |
|  |  | 0.159 | 1.153 | 1.713 | 0.224 | > 100 |  |  |  |  |
| ***CI*** |  | -0.016 | -0.115 | -0.12 | -0.053 | 0.539 | 0.519 |  |  |  |
|  |  | 0.117 | 0.788 | 0.927 | 0.171 | > 100 | > 100 |  |  |  |
| ***PC*** |  | -0.068 | -0.062 | -0.11 | -0.058 | 0.652 | 0.641 | 0.628 |  |  |
|  |  | 0.223 | 0.198 | 0.654 | 0.186 | > 100 | > 100 | > 100 |  |  |
| ***IB*** |  | -0.051 | -0.08 | -0.103 | -0.063 | 0.68 | 0.671 | 0.594 | 0.748 |  |
|  |  | 0.164 | 0.285 | 0.527 | 0.2 | > 100 | > 100 | > 100 | > 100 |  |
| ***SOGS*** |  | 0.034 | -0.166 | -0.146 | -0.104 | 0.513 | 0.625 | 0.436 | 0.547 | 0.582 |
|  |  | 0.133 | 6.559 | 2.603 | 0.544 | > 100 | > 100 | > 100 | > 100 | > 100 |

**Figure A1.** Violin/box plots for age, education years, matrices scores, GRCS subscores, and SOGS severity scores, and frequency histogram for the number of correct responses in the BNT, across groups (PGD: patients with gambling disorder; NPG: Individuals in non-problematic gambling).

| Age  INCLUDEPICTURE "http://127.0.0.1:49622/9943cc26-9d5a-4d49-b001-7a2abfc4824b/2/res/02 descriptives/resources/02ba0149c782a0f0.png" \* MERGEFORMATINET 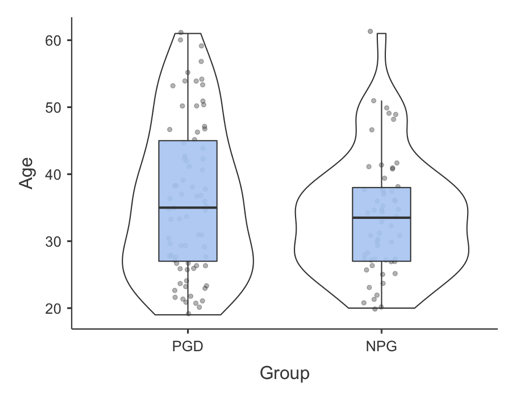 | Education  INCLUDEPICTURE "http://127.0.0.1:49622/9943cc26-9d5a-4d49-b001-7a2abfc4824b/2/res/02 descriptives/resources/408f609249351c60.png" \* MERGEFORMATINET 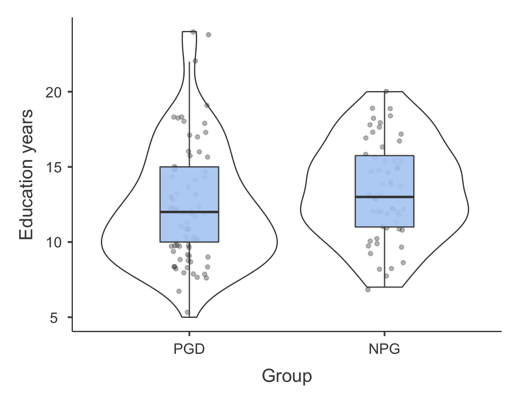 | |
| --- | --- | --- |
| Matrices  INCLUDEPICTURE "http://127.0.0.1:49622/9943cc26-9d5a-4d49-b001-7a2abfc4824b/2/res/02 descriptives/resources/b80915c1d18e1c72.png" \* MERGEFORMATINET 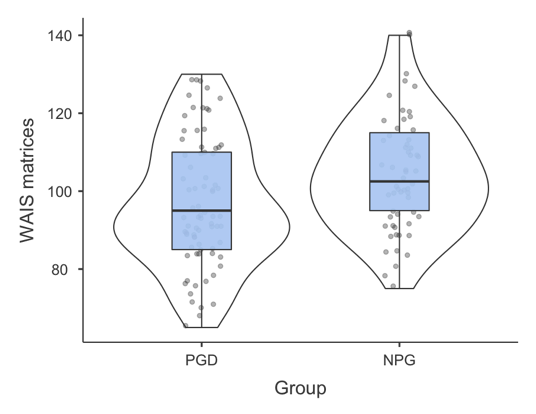 | BNT  INCLUDEPICTURE "http://127.0.0.1:49622/9943cc26-9d5a-4d49-b001-7a2abfc4824b/2/res/02 descriptives/resources/29ae633819067f1b.png" \* MERGEFORMATINET 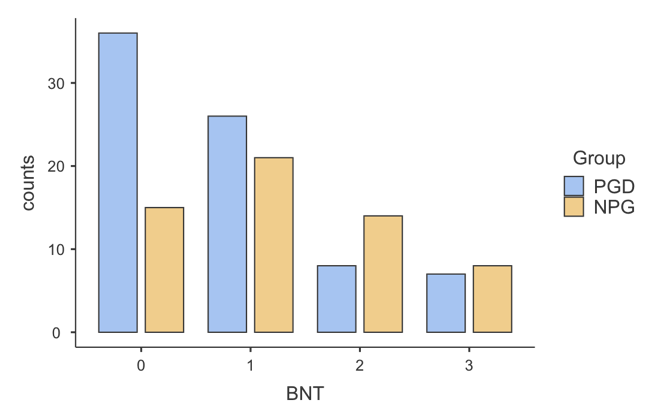 | |
| Expectancies  INCLUDEPICTURE "http://127.0.0.1:49622/9943cc26-9d5a-4d49-b001-7a2abfc4824b/2/res/02 descriptives/resources/71482c4b26d179aa.png" \* MERGEFORMATINET 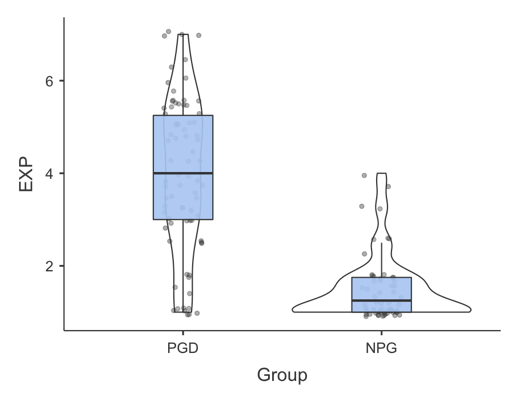 | Inability to stop  INCLUDEPICTURE "http://127.0.0.1:49622/9943cc26-9d5a-4d49-b001-7a2abfc4824b/2/res/02 descriptives/resources/57173d81858cdcde.png" \* MERGEFORMATINET 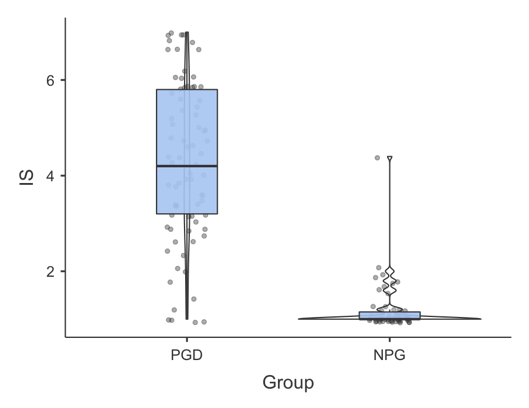 | Control Illusion  INCLUDEPICTURE "http://127.0.0.1:49622/9943cc26-9d5a-4d49-b001-7a2abfc4824b/2/res/02 descriptives/resources/088375a4052dc4d2.png" \* MERGEFORMATINET 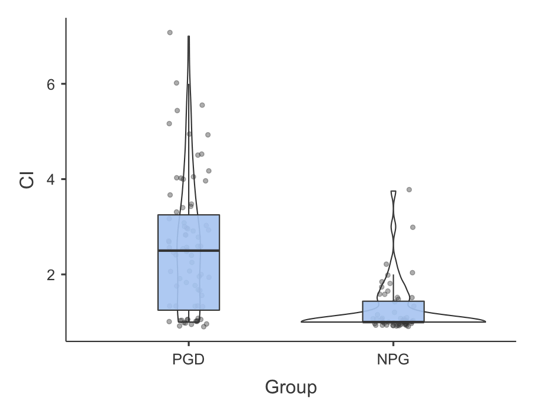 |
| Predictive control  INCLUDEPICTURE "http://127.0.0.1:49622/9943cc26-9d5a-4d49-b001-7a2abfc4824b/2/res/02 descriptives/resources/b25ea8d2e03d4333.png" \* MERGEFORMATINET 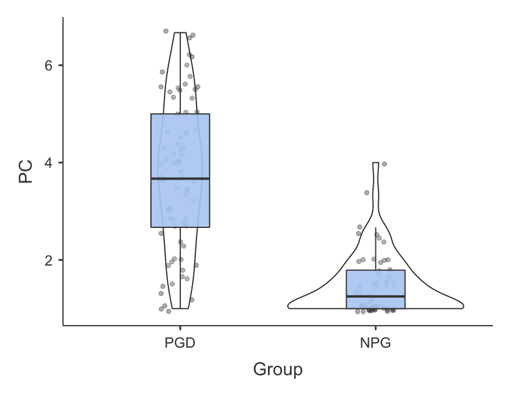 | Interpretative bias  INCLUDEPICTURE "http://127.0.0.1:49622/9943cc26-9d5a-4d49-b001-7a2abfc4824b/2/res/02 descriptives/resources/bd5dd49507c8f8ae.png" \* MERGEFORMATINET 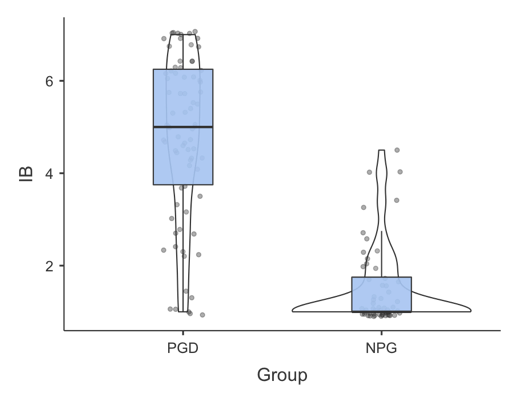 | SOGS  INCLUDEPICTURE "http://127.0.0.1:49622/9943cc26-9d5a-4d49-b001-7a2abfc4824b/2/res/02 descriptives/resources/3c024029d43bd46b.png" \* MERGEFORMATINET 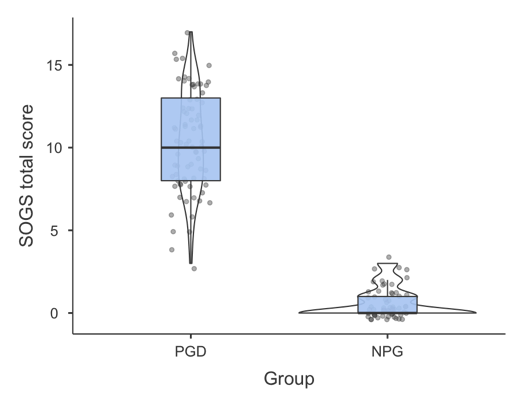 |

**Figure A2. Predicted total GRCS scores as a function of BNT scores, matrices scores, age and education. Reference values for age and education years were selected as +1, 0, and -1 standard deviations.**

| **BNT interaction with age**  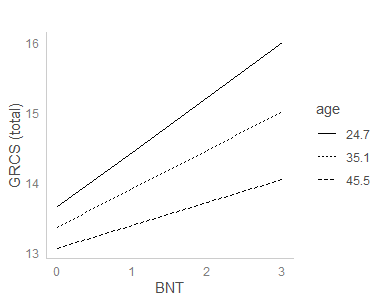 | **BNT interaction with education**  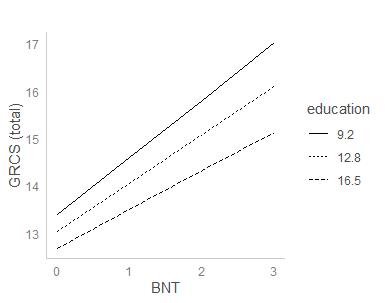 |
| --- | --- |
| **Matrices interaction with age**  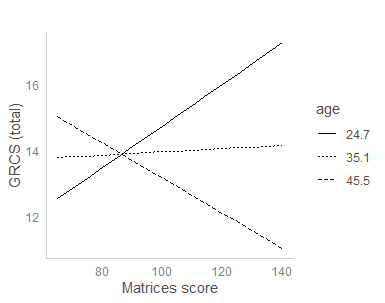 | **Matrices interaction with education**  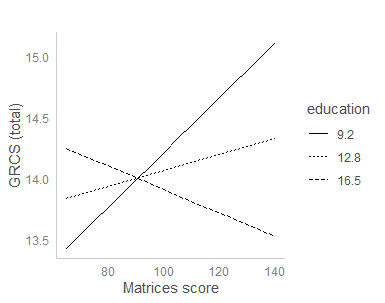 |

**Figure A3.** Graphic depiction of the correlation matrix for all variables of interest, collapsing groups (Matrix: WAIS matrices scores, BNT: Berlin Numeracy Test, EXP: Gambling Expectancies, IS: Inability to Stop, CI: Control Illusion, PC: Predictive Control, IB: Interpretative Bias, SOGS: Gambling severity).


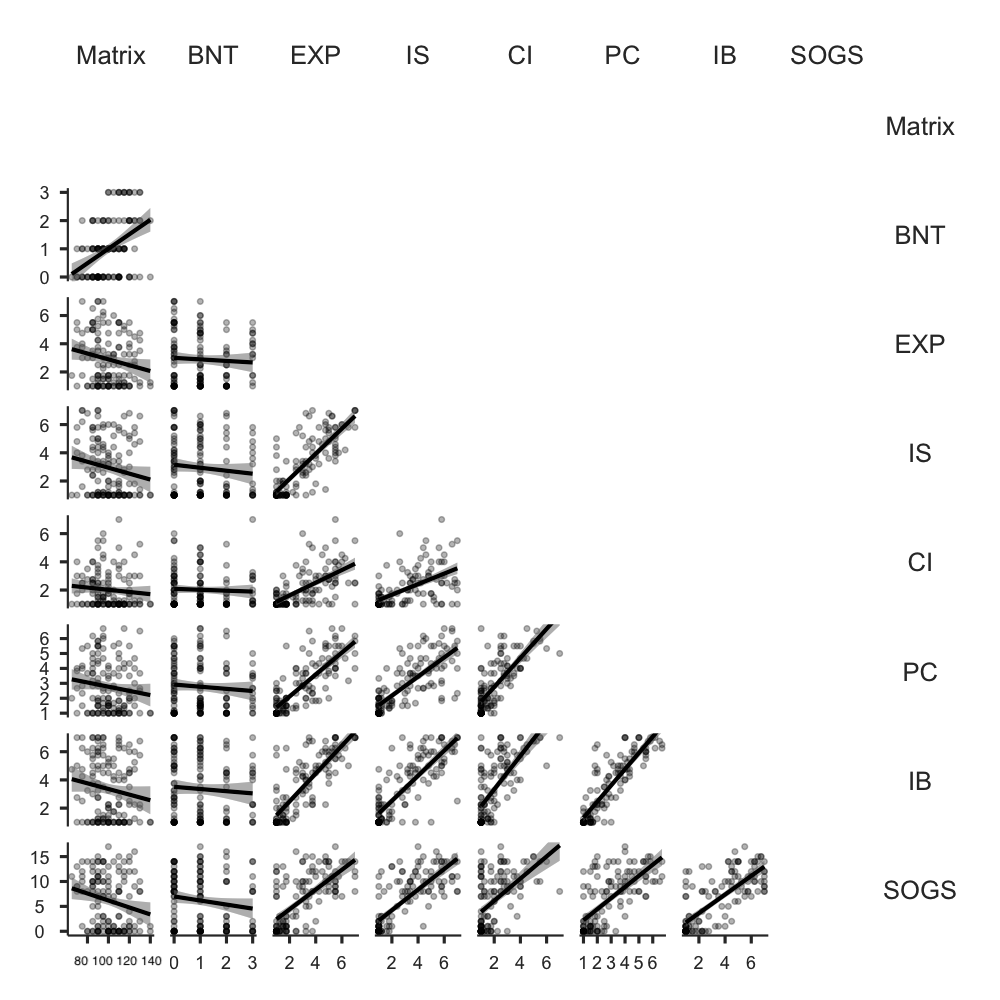

Supplement: Supplementary file 1 [file Table_1.DOC]
